# Flipping for success: evaluating the effectiveness of a novel teaching approach in a graduate level setting

**DOI:** 10.1186/s12909-015-0317-2

**Published:** 2015-02-28

**Authors:** John Moraros, Adiba Islam, Stan Yu, Ryan Banow, Barbara Schindelka

**Affiliations:** 1 School of Public Health, University of Saskatchewan, 104 Clinic Place, E-Wing Health Sciences, Room 3320, Saskatoon, SK S7N 2Z4 Canada; 2 School of Public Health, University of Saskatchewan, Saskatoon, Canada; 3 Research and Program Evaluation Analyst, Gwenna Moss Centre for Teaching Effectiveness, University of Saskatchewan, Saskatoon, Canada; 4 Instructional Design Specialist, Gwenna Moss Centre for Teaching Effectiveness, University of Saskatchewan, Saskatoon, Canada; 5 Instructional Design Specialist, Gwenna Moss Centre for Teaching Effectiveness, University of Saskatchewan, Saskatoon, Canada

**Keywords:** Flipped Classroom, Education, Instructional technology, Students, Graduate level setting

## Abstract

**Background:**

Flipped Classroom is a model that’s quickly gaining recognition as a novel teaching approach among health science curricula. The purpose of this study was four-fold and aimed to compare Flipped Classroom effectiveness ratings with: 1) student socio-demographic characteristics, 2) student final grades, 3) student overall course satisfaction, and 4) course pre-Flipped Classroom effectiveness ratings.

**Methods:**

The participants in the study consisted of 67 Masters-level graduate students in an introductory epidemiology class. Data was collected from students who completed surveys during three time points (beginning, middle and end) in each term. The Flipped Classroom was employed for the academic year 2012–2013 (two terms) using both pre-class activities and in-class activities.

**Results:**

Among the 67 Masters-level graduate students, 80% found the Flipped Classroom model to be either somewhat effective or very effective (M = 4.1/5.0). International students rated the Flipped Classroom to be significantly more effective when compared to North American students (X^2^ = 11.35, p < 0.05). Students’ perceived effectiveness of the Flipped Classroom had no significant association to their academic performance in the course as measured by their final grades (***r*s** = 0.70). However, students who found the Flipped Classroom to be effective were also more likely to be satisfied with their course experience. Additionally, it was found that the SEEQ variable scores for students enrolled in the Flipped Classroom were significantly higher than the ones for students enrolled prior to the implementation of the Flipped Classroom (p = 0.003).

**Conclusions:**

Overall, the format of the Flipped Classroom provided more opportunities for students to engage in critical thinking, independently facilitate their own learning, and more effectively interact with and learn from their peers. Additionally, the instructor was given more flexibility to cover a wider range and depth of material, provide in-class applied learning opportunities based on problem-solving activities and offer timely feedback/guidance to students. Yet in our study, this teaching style had its fair share of challenges, which were largely dependent on the use and management of technology. Despite these challenges, the Flipped Classroom proved to be a novel and effective teaching approach at the graduate level setting.

## Background

The face of higher education among the health professions is ever changing and constantly evolving. A growing body of literature suggests that the use of digital educational technologies is rapidly expanding in this arena and it is becoming a high priority for many academic institutions of higher learning [1-3]. Advances in technology have led to a number of blended learning initiatives (which combine classroom and online education) across the globe and especially in North America [4,5]. These initiatives are now seen as playing an increasingly more influential role in the way today’s health professions students assimilate information and learn within an educational setting. It has been suggested that blended learning can help maximize instructor efficiency, increase student engagement, reach more students, and improve retention rates [6]. One of the applications of blended learning that has gained popularity over the past few years is the Flipped Classroom.

The Flipped Classroom is an educational model in which the standard lecture and homework elements of a course are “reversed” or “flipped”. [7] Flipped Classroom often involves students viewing pre-recorded lecture videos prior to attending class and using the class time to engage in student-centered learning activities like inquiry and problem solving but it may take on many different forms. The goal of most applications of the Flipped Classroom is to provide an opportunity for students to read/view course related material at their own pace and on their own time prior to the actual class. Once they arrive to class, they are now ready to apply this new found knowledge through problem based learning exercises in order to facilitate their critical thinking and deep learning of the subject matter.

The Flipped Classroom has underpinnings in both the constructivist [8] and social learning theory [9] because it permits and encourages students to view learning as an active and social process. In this manner, while students receive mentored guidance from their instructor, they are allowed to use their “learning-by-doing” experiences to help construct, organize and support their own knowledge and educational advancement. By comparison, traditional lecture courses can be quite limiting because often times, they do not provide sufficient face-to-face time for students to apply course related material in the class. Therefore, students are required to complete this deeper learning on their own, after a lecture is given, and without the guidance and support of their instructor or peers.

The Flipped Classroom approach also provides instructors with pedagogical latitude to implement a wide range of constructivist and creative social learning activities during dedicated class time that may prove beneficial to the students. Additionally, the instructor now liberated from the purely didactic responsibilities of the course can use the classroom time to help guide students through interactive, collaborative exercises that require higher level critical thinking and reasoning skills. These exercises are designed to assist the students attain higher educational outcomes within Bloom’s Taxonomy [10]. It has been noted that these types of cognitive skills are fundamentally important in the career preparation and ultimate success of students in the health professions [11].

Despite its considerable publicity and obvious advantages, the Flipped Classroom approach to date has garnered limited scholarly research in its use in higher education and particularly in its effectiveness. Much of the use of the Flipped Classroom has primarily concentrated in K-12 [12] and secondarily in undergraduate educational settings [13]. A review of the limited research literature reveals that students may have mixed views on the Flipped Classroom. In some studies, students have found the Flipped Classroom to be relative superior to the traditional lecture approach [4,14], whereas in others, students have reported lower levels of satisfaction [15]. Yet, other studies have mainly examined the Flipped Classroom within the context of student achievement rather than satisfaction [16]. In a study conducted by Zappe and colleagues, the findings suggest that although students found benefit in the Flipped Classroom approach, they would prefer only about half of the class sessions to be “flipped” and the others to be provided in a “traditional” setting [17].

Thus, while some important work has been conducted in this area, there still remains a significant void in research of the Flipped Classroom approach. This study builds on and further expands our collective knowledge in the field as it examines the use and effectiveness of the Flipped Classroom approach in a graduate-level, introductory epidemiology course setting. The purpose of this study is four-fold and aims to compare Flipped Classroom effectiveness ratings with: 1) student socio-demographic characteristics, 2) student final grades, 3) student overall course satisfaction, and 4) course pre-Flipped Classroom effectiveness ratings.

## Methods

### Participants

There were 76 Masters of Public Health students enrolled in an introductory, graduate level epidemiology course during the academic year 2012–2013. Of the 76 students, 67 of them (88%) agreed to participate in this study. Almost half of the participants (49.3%) were 25 years old or younger and many of them represented a group of students that had continuously transitioned academically from high school to college to graduate school. Whereas the other half of the study participants were 26 years old and older and therefore, many of them represented a group of students that upon completion of their undergraduate studies had sought and gained work related experiences and then decided to come back and pursue graduate school studies in order to further their professional careers. The majority of the participants indicated that they were either comfortable or very comfortable with using computers and the internet (89%) as well as the Blackboard Learning System (89%). The key participant demographic information is presented in Table 1.

**Table 1:** **Participant students’ demographic information**

Characteristics		(N = 67)
**Gender**	Female	61.2%
	Male	38.8%
**Age**	≤25 years old	49.3%
	26-30 years old	31.3%
	>30 years old	19.4%
**Highest level of previous education**	Bachelor’s	89.6%
	Master’s	6.0%
	Doctorate	4.5%
**Location of previous education**	North America	61.2%
	Outside North America	38.8%
**Level of comfort with using computers and the Internet**	Comfortable or very comfortable	89.0%
**Level of comfort with using the Blackboard learning management system**	Comfortable or very comfortable	89.0%

### Rational for the Flipped Classroom

The course was officially “flipped” in the fall of 2012, after two years of careful deliberations and strategic planning among members of the research team. During this time, the instructor entered into 10 bi-monthly, in-depth consultations with the teaching and learning centre staff and extensively familiarized himself with all relevant literature. Additionally and on the basis of qualitative feedback provided to the instructor by graduating students, it was determined that redesigning the class along the Flipped Classroom model will not only improve the educational experience of our students but it will also satisfy their stated desire to engage in more practical, hands-on, in-class learning activities. This was well in line with the instructor’s overarching goal to actively engage graduate students through the use of creative technologies and applied learning in a collaborative setting so as to train them to become critical thinkers and real life, problem solvers.

### Structure and settings of the Flipped Classroom

The Flipped Classroom was employed during the academic year 2012–2013 (two terms) using both pre-class activities (online video lectures and textbook readings) and in-class activities (quizzes, practice problem sets, and student presentations). The class met once a week for three hours for 13 weeks, each term.

For the pre-class activities, students were asked to view the pre-recorded video lectures from the instructor prior to coming to class. There was one video for every in-class session and they averaged approximately 60 minutes in length. The videos were viewable on desktop or laptop computers but not on mobile devices. Students were also expected to read the corresponding textbook chapters. Pre-class activities were strongly recommended but voluntary.

The in-class activities began with a short quiz (10–15 minutes) that tested the students’ understanding and knowledge of the information presented in the corresponding pre-recorded video and textbook readings. The quiz was completed by each individual student and consisted of a mixture of true or false, matching, short answer, workout the problem and multiple-choice questions. In total, there were 10 quizzes given each term. These quizzes were graded in class by a Teaching Assistant (TA) and returned to the students by the end of the class to provide immediate feedback. Following the quiz, the instructor led a review of the quiz questions through an open discussion forum, soliciting the right answers as well as additional perspective from the students.

Afterwards, the instructor presented a short but in depth lecture on the corresponding topic. These short lectures were usually 20–30 minutes in length and were used by the instructor in order to provide clarity of difficult concepts and reinforce the students’ learning. Later, the class was divided into their pre-assigned small groups (three to four students per group) so as to collaboratively work on solving relevant practice problems. These sessions were usually 45–60 minutes long and permitted students to share their ideas, learn from each other, and work collaboratively in solving practice problems. Students from different groups were asked by the instructor to come to the whiteboard and present their solutions to the practice problems to the rest of the class. If there was lack of consensus as to the right solution for a particular problem then the instructor followed up with the right solution while providing a rational for it and generating guided feedback to ensure appropriate learning.

**Figure 1:**
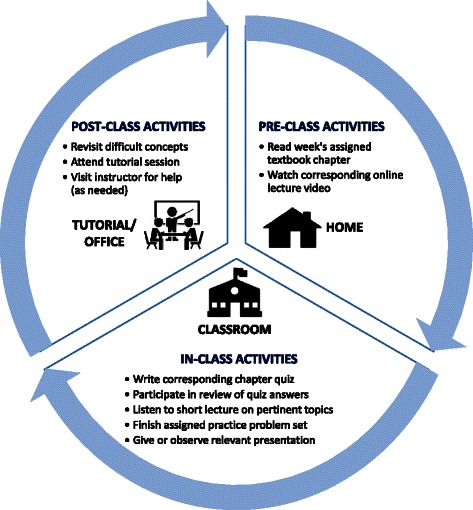
**Flowchart of the Flipped Classroom structure and settings.**

The final portion of the class was devoted to student presentations. Each week, a pre-assigned group (three to four students per group) was responsible for presenting and answering follow up questions with regard to recent (last five years), peer reviewed article from the primary literature related to that specific week’s class topic. There was an anonymous peer evaluation sheet completed by each non-presenting student that was submitted to the instructor immediately following the group presentation. At the end of the class, the presenting group met and de-briefed with the instructor. The group was provided the comments from their peers and more structured feedback by the instructor, who also assigned them a grade. Each group presented twice each term and all students in each group received the same grade. All in-class activities were mandatory (Figure 1).

### Assessment of the Flipped Classroom

At the beginning of each term, a course syllabus was provided to the students that concisely introduced them to the nuances of the Flipped Classroom while clearly articulating the assessment aspects for the course. In this study, the Flipped Classroom used both a formative and summative assessment of students’ learning.

The formative assessment was predominantly centered on the in-class activities as articulated in the preceding section. These in-class activities permitted students to be purposively engaged in their own learning while it enabled the instructor and TA to provide real-time guidance and feedback, and as appropriate fill in the students’ gaps in knowledge. The formative assessment was comprised of 10 quizzes (worth 1% each for a total 10%) and 2 student group presentations (worth 5% each for a total of 10%).

The summative assessment was comprised of both take home and in-class activities. The take home activities included two major assignments (worth 10% each for a total of 20%) that provided additional practice and more advanced learning opportunities for students. The in-class activities included a midterm (20%) and a final exam (40%). The assignments and exams assessed the students’ ability to critically read, extract, tabulate, analyze and interpret information and encouraged them to use higher order critical thinking and reasoning skills.

### Data collection and instruments

Surveys were administered to students and data collected during three separate time points in each term (during the first week of the class or ‘the first time point’, at the midpoint of the class or ‘the second time point’, and at the last day of the class or ‘the third time point’). Participation in the study was voluntary with no tangible incentives provided to the students. Participant anonymity was maintained throughout the study by assigning each student a survey code at the first time point by the non-teaching members of the research team. This same code was used again for the second and third time points. Each student gave written, informed consent before their participation in the study. The Behavioral Research Ethics Board at the University of Saskatchewan reviewed and approved this study (BEH#12-257).

The first time point consisted of one survey. This first survey collected student demographic data and initial thoughts about learning technologies, course content and the Flipped Classroom. The survey contained 22 questions in total, 17 of which were selected response and five were open-ended questions. The second time point also consisted of one survey. This survey was very similar to the first survey, except it did not contain the demographics questions. It had 18 questions in total, 13 of which were selected response and five were open-ended questions. The third time point consisted of two surveys: 1) a survey that was identical to the one used in the second time point and 2) the Student Evaluation of Educational Quality (SEEQ) course survey.

SEEQ is a standardized instrument that is used by the University of Saskatchewan to obtain student feedback on teaching quality and effectiveness. SEEQ was developed by educational psychologist Herbert Marsh, and is one of the most widely used and empirically supported evaluative instruments in post-secondary institutions. It is comprised of items grouped into eight distinct dimensions of teaching (Learning, Enthusiasm, Organization, Group Interaction, Individual Rapport, Breadth, Examinations, and Assignments) [18].

The study surveys mainly used Likert-scale questions that asked students to rate the effectiveness of different elements of the Flipped Classroom. Students rated elements, such as “Working through practice problem-solving questions together in class”, on a scale of 1 to 5 (1 = very ineffective, 2 = somewhat ineffective, 3 = neither effective nor ineffective, 4 = somewhat effective, and 5 = very effective). The open-ended questions explored general thoughts by students on the use of learning technologies and the Flipped Classroom. The same survey questions were asked at all three time points in the academic term so as to be able to compare changes in student perceptions of the effectiveness of the Flipped Classroom.

## Results

### Overall effectiveness ratings from students

The study measured the students’ perceived effectiveness of the Flipped Classroom at three distinct time points in the term and the extent to which their perceptions changed, either positively or negatively, during the duration of their experience in the classroom (Table 2). Repeated-measures ANOVAs were conducted to estimate the change within participants’ perceptions at all three time points of the study. Since students’ ratings for quizzes and group presentations were not asked in the first time point, non-parametric Wilcoxon-signed rank tests were used for analysis of the second and third time point comparisons.

**Table 2:** **Students’ ratings of the effectiveness of the Flipped Classroom**

Please rate your general effectiveness of:	Mean		
	First point	Second point	Third point
	(s.d.)	(s.d.)	(s.d.)
In-person lectures	4.27/5.00 (0.73)	3.98/5.00 (1.1)	4.07/5.00 (1.1)
Practice problem-solving questions together in class	4.55/5.00 (0.6)	4.37/5.00 (1.0)	4.62*/5.00 (0.7)
Class or Group discussions	4.25/5.00 (0.8)	4.25/5.00 (0.9)	4.27/5.00 (0.9)
Applying concepts to real-life case studies or situations	4.57/5.00 (0.6)	4.23/5.00 (1.0)	4.43/5.00 (0.8)
Video lectures	3.90/5.00 (0.9)	3.56/5.00 (1.2)	3.66/5.00 (1.1)
Quizzes	N/A	4.39/5.00 (0.8)	4.59*/5.00 (0.8)
Group presentations	N/A	3.77/5.00 (1.0)	3.66/5.00 (1.1)

*Significant at 0.05; n = 60; Likert scale: 1 = very ineffective to 5 = very effective.

N/A = Not Applicable.

Overall, it was found that students entered into the class with high expectations for how effective they felt the Flipped Classroom would be, as evidenced by ratings ranging from 4.2- 4.6/5.0 on four of the five items considered on the first time point. However by the second time point, students’ expectations dipped and they rated four of the five components of the Flipped Classroom lower when compared to the first time point. At the third time point, a general increase in students’ ratings of the effectiveness of the Flipped Classroom was observed when compared to the second time point. Interestingly, students rated the effectiveness of working collaboratively on problem-solving activities in class and class/group discussions significantly higher in the third time point than both the first and second time points.

Repeated measures ANOVA showed that students’ ratings of the effectiveness of working collaboratively on problem-solving activities in class differed significantly between the three time points, V = 0.1, *F*(2, 58) = 3.23, p < 0.05. Post-hoc tests also revealed that students were significantly more likely to rate the in-class activities as being more effective in the post-term survey (i.e. third time point) in comparison to the midpoint of the class (i.e. second time point). Meanwhile, at the third time point, students rated the elements of the in-person lecture, applying concepts to real-life studies, and video lectures higher in comparison to the second time point, though their ratings still remained lower than the ones recorded in the first time point.

For the two elements of the Flipped Classroom that were only asked in the second and third time points, it was found that students were significantly more likely to rate the effectiveness of quizzes higher at the third time point in comparison to the second time point (*z* = −3.36, p < 0.05). Conversely, students rated the effectiveness of the group presentations to be marginally less effective in the third time point when compared to the second time point.

**Figure 2:**
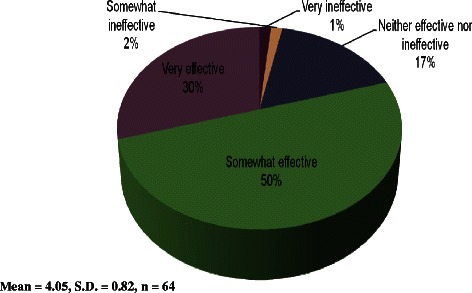
**Students’ overall effectiveness rating of the Flipped Classroom.**

The core elements of the Flipped Classroom, consisting of the in-person lecture, working through practice problem-solving questions together in class, class/group discussions, applying concepts to real-life case studies or situations and quizzes, were summated to create a student rating of the Flipped Classroom’s overall effectiveness. Figure 2 illustrates the distribution of these responses. The majority of students (80%) found the Flipped Classroom to be either somewhat effective or very effective with an overall mean of 4.1/5.0.

### Comparing effectiveness ratings from international vs. North American students

**Figure 3:**
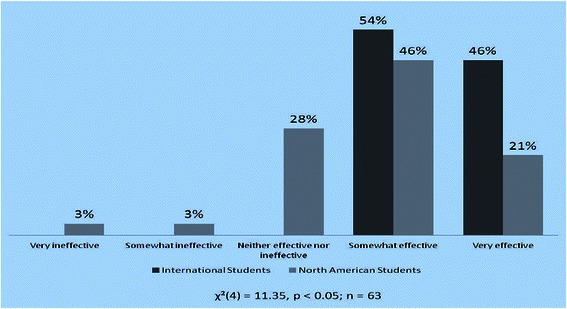
**Comparing International and North American students’ effectiveness ratings of Flipped Classroom.**

There were 24 International graduate students who participated in the study and all of them (100%) found the Flipped Classroom to be either somewhat effective or very effective. By comparison, this sentiment was shared by only 67% of North American graduate students (Figure 3). The results of chi-square test analysis revealed that International students found the Flipped Classroom to be significantly more effective in comparison to North American students.

### Comparing effectiveness ratings from students with final grades

Spearman’s correlations were used to investigate whether an association existed between students’ overall rating of the effectiveness of the Flipped Classroom and the final grade they attained in the class. Interestingly, we found that students’ perceived effectiveness of the Flipped Classroom had no significant association to their academic performance as measured by their final grade (***rs*** = .70).

### Comparing effectiveness ratings from students with overall course satisfaction

Additionally, we assessed whether an association existed between students’ overall rating of the Flipped Classroom effectiveness and their satisfaction ratings on SEEQ course survey. As the SEEQ instrument organizes its individual items into subsets with each set measuring a particular variable of teaching, the individual items on SEEQ were summated into their respective SEEQ variables. Correlations were conducted between students’ perceived effectiveness of the Flipped Classroom with SEEQ variables. Table 3 shows that a positive and moderate correlation exists between students’ rating of the overall effectiveness of the Flipped Classroom and all of the SEEQ variables. In other words, students who found the Flipped Classroom effective were also more likely to report a higher overall satisfaction for the course. All correlations were significant at the 0.001 level.

**Table 3:** **Spearman correlations between students’ overall Flipped Classroom effectiveness score and SEEQ variables**

	SEEQ learning	SEEQ enthusiasm	SEEQ organization	SEEQ group interaction	SEEQ individual rapport	SEEQ breadth	SEEQ examinations	SEEQ assignments
Overall effectiveness rating of the Flipped Classroom	.590*	.604*	.635*	.503*	.528*	.608*	.503*	.489*

*p < 0.001.

### Comparing effectiveness ratings pre- and post-flipped classroom implementation

Finally, a paired samples t-test was conducted to compare the SEEQ variable scores between the students in this sample (n = 60) and the scores of students enrolled in the year prior to the implementation of the Flipped Classroom model (n = 52). In turn, it was found that the SEEQ variable scores for students enrolled in the Flipped Classroom (M = 4.47, SD = 0.12) were significantly higher than the SEEQ variable scores for students enrolled prior to the implementation of the Flipped Classroom (M = 3.82, SD = 0.4, t(8) = −6.46, p = 0.003). While this finding suggests an overall higher level of course satisfaction amongst students that had experienced the Flipped Classroom pedagogical model than their respective cohort that had experienced traditional lecturing, it is important to note that this comparison did not control for other factors that may have accounted for this difference.

## Discussion

Evaluating the effectiveness of the Flipped Classroom is important in furthering our understanding of the use and impact this novel teaching approach may have in a graduate level setting. In order for students to experience success, the Flipped Classroom method requires that students first complete their assigned readings and carefully review the preparatory material (video lectures) prior to attending class [14]. Only then can the students’ learning be maximized during in-class time because the topics covered often build upon one another incrementally and course examinations are heavily dependent on higher order thinking and reasoning skills.

In this study, the overall effectiveness of the Flipped Classroom method of teaching was rated high by students. Its central lure was the fact that pre-class, students were able to work at their own pace while in-class, there was more time to practice and collaboratively participate in applied “homework” related activities and gain better familiarity with potential “test material” by way of the weekly quizzes. In general, students felt they were given a greater number of opportunities to be actively engaged in their own learning and progressively improve their mastery over the course content. As stated in previous studies, the Flipped Classroom is a teaching method that promotes student thinking both inside and outside of the classroom [19].

However, while the in-class practice problem sets and quizzes were found to be valuable to students, the group presentations and video lectures were found to be less so (Table 2). There were a number of well-reasoned explanations for this finding. For the group presentations, some students had a problem with the fact that individual members in any given group were not held accountable for their degree of involvement/contribution because the final mark was a collective group mark. Some students suggested incorporating an evaluation measure for their individual team members in order to allow for increased accountability. In other cases, students commented that they did not particularly enjoy the group presentations because presenting was considered to be an “uncomfortable” and in some instances a “frightening” experience.

Another noteworthy finding was based on the fact that some students reported the video lectures to be limited in several technological aspects. Some of the videos were found to be of poor audio quality, too long in duration, and did not allow for immediate feedback. These findings are not surprising as previous studies have also highlighted the limitations of technology as an important factor in determining the student experience in the Flipped Classroom [15].

A further point of interest was examining whether International students were more likely to find higher value in the pedagogical approach provided by the Flipped Classroom when compared to North American students (Figure 3). One of the key advantages of the Flipped Classroom is that it allows students to move through content and learn at their own individual pace [14]. In our study, this proved to be of significant value to International students, who may experience language barriers and difficulties in comprehension of complex concepts, unlike their North American counterparts.

When given the option to view video lectures outside of class, International students were given in effect the opportunity to ‘pause’ and ‘rewind’ the subject matter at their discretion, which would otherwise not be possible in a traditional lecture setting. As such, if they have difficulties understanding what the instructor is saying or if they are struggling with the fast pace of the course, they can revisit the video lecture or certain parts of it, as many times as they desire, until they are able to comprehend the topics being covered to their satisfaction. This is an important pedagogical consideration for International students for whom English is their second language. The use of the Flipped Classroom can help ‘level the playing field’ and make a significant impact on their ability to overcome language barriers critical to their learning and academic success.

Previous studies have hypothesized that students who performed well academically in a particular course were more likely to positively evaluate their instructor [20,21]. However, the findings from our study indicate that the students’ academic performance did not influence their perception of how effectively the course was taught – the two variables were independent. As such, students in this study exhibited a well-reasoned approach in distinguishing between their individual academic performance and how they valued and assessed the effectiveness of their learning environment in the course (i.e. Flipped Classroom).

Academic performance has many different measures, besides a student’s final grade [22]. It is entirely possible that the overall effectiveness of the Flipped Classroom lies in the fact that it promotes higher order thinking, learning, and mastery of the subject matter on a consistent basis and throughout the course when compared to a traditional lecture format. In a traditional lecture, students usually have the tendency to study more heavily just prior to the midterm and final examinations. On the other hand, in the Flipped Classroom, students are strongly encouraged to study and learn the subject matter more regularly since they get tested on a weekly basis (i.e. by way of quizzes and in-class practice problem sets).

In this study, the Flipped Classroom provided the students and the instructor with multiple educational advantages. From the students’ perspective, it increased flexibility in learning because it allowed them to progress at their own pace (i.e. replay the lecture videos as many times as needed to better understand key concepts) and it increased free class time to practice and master applied skills (i.e. problem-solving activities). Additionally, students were permitted to further their understanding by critically thinking about, actively discussing and more importantly, peer teaching key concepts in a collaborative classroom setting. In this manner, the Flipped Classroom made learning more manageable for students by taking difficult tasks and complex ideas and making them more understandable and accessible.

From the instructor’s perspective, this setting made it easy to engage students and empower them to become active participants in their own learning. The Flipped Classroom not only permitted the instructor to provide the students with a wider breath and deeper understanding of the material covered but having more collaborative activities take place during class helped built cohort comradery and generate much enthusiasm for learning by the students (Table 3). Finally, the Flipped Classroom allowed the instructor to gain advanced, real-time insight into how students learn and quickly identify and better address curriculum content the students found to be most challenging. This insight can be used to better inform decisions with regard to effective curriculum organization, structuring and delivery of future classes.

### Limitations

This study does have several limitations. First, no control or comparison groups were used during the time period under investigation so as to concurrently compare the effectiveness of the Flipped Classroom between an “experimental” and a “control” group. However, course data on the course effectiveness ratings had been collected by way of SEEQ evaluations both pre- and post-Flipped Classroom implementation and were used in this study. Second, the instruments developed for the Flipped Classroom survey were newly designed and specifically tailored to this course. Therefore, they lack evidence of reliability and external validity. Finally, the findings of this study may not be generalizable to other graduate level courses and/or higher institution settings.

### Challenges

The main challenges that arose from using the Flipped Classroom approach in a graduate level setting were two-fold and included issues due to student comfort level and use of technology. While managing technology was a big issue in our administration of the Flipped Classroom, another equally important aspect of this teaching model is the need to ensure that students are actually stimulated in class and find the learning environment to be safe (without discomfort and fear), supportive and beneficial to their learning.

On the technology front, students found the audio quality of certain videos to be poor. On those occasions, it made it difficult for students to clearly hear the instructor irrespective of how high they adjusted their audio settings (Table 2). They also commented on how some of the video lectures had background noise, which made it difficult for them to easily follow along and fully comprehend the concepts being explained by the instructor. In other instances, students were not pleased with administrative processes that resulted in the delay by a few days of the release time of certain videos. They felt that the lack of timely distribution of the video lectures did not allow them sufficient time to adequately prepare for the quizzes and the in-class practice problems, which were issued on a weekly basis.

Moreover, when they did view the video lectures at home, some students expressed dissatisfaction with the fact that they could not ask questions of the instructor in real-time. The instructor had recommended to students to record their questions during their viewing of the video and bring them to the next tutorial session and/or class as an item for discussion but some students felt that the lag in time did not appropriately facilitate their learning. Also, a few students stated that by the time they arrived to the tutorial session and/or the class, they had forgotten which areas they had difficulties understanding in the videos.

The final concern expressed by some students was the fact that the video lectures were often over an hour long in length, which made it difficult for them to view them in a single seating. Further complicating this issue was the fact that on those occasions when students decided to pause the video and return to it a short time later, many experienced technical difficulties. They were unable to recommence viewing the video from the point where they had previously paused it since the video location had returned to its original starting point.

### Future directions

Creating an effective and sustainable learning environment requires constant monitoring and timely adjustments. On the basis of the feedback provided to the research team by our students, we have identified several areas that require further improvement. First, the video lectures need to be significantly modified and a different, more user-friendly recording platform needs to be seriously considered. Second, the videos should be broken down into multiple, shorter video file segments (i.e. 2–3 videos of 20 to 30 minutes length each) so that students are able to give their undivided attention and fully concentrate on the content presented in one sitting. Additionally, the shorter videos will permit students to more easily pause and return to their video as well as allow them to watch each section at different times of the week, depending on their schedule. Third, the audio quality of the videos needs to be dramatically improved by making every effort to remove/eliminate the background noise. Fourth, an online forum needs to be created so as to permit the instructor and/or the TA to directly communicate with the students and address any pertinent questions in a timely manner prior to attending the actual class. Finally, administrative barriers need to be removed and more autonomy afforded to the instructor in order to ensure the timely release of the videos to the students.

### Recommendations

In summary, the following recommendations may prove of use and benefit to other instructors who may be contemplating using the Flipped Classroom approach in their own class. First, make certain to create a safe and supportive “blended” learning environment for all your students. Second, provide any IT support required by your students to be able to regularly access and view the video lectures. Third, require students to complete the weekly quiz individually and in-class so as to ensure that each one of them comes to class well prepared and ready to engage in deep learning. Fourth, keep the videos relatively short (no longer than 20–30 minutes) to facilitate the students learning and ensure that they watch them. Fifth, create an online forum so as to permit the students to directly communicate with the instructor and/or the TA and be able to post and receive answers to pertinent questions prior to attending class. Finally, encourage your students to use multi-media aspects in their own class related presentations so they may gain increased comfort, familiarity and confidence in using the technology, and therefore, be more likely to regularly use it within the broader structure of the Flipped Classroom.

## Conclusions

Overall, the format of the Flipped Classroom provided more opportunities for students to engage in critical thinking, independently facilitate their own learning, and more effectively interact with and learn from their peers. Additionally, the instructor was given more flexibility to cover a wider range and depth of material, provide in-class applied learning opportunities based on problem-solving activities and offer timely feedback/guidance to students. Yet in our study, this teaching style had its fair share of challenges, which were largely dependent on the use and management of technology. Despite these challenges, the Flipped Classroom proved to be a novel and effective teaching approach at the graduate level setting.

## Abbreviation

SEEQ: Student Evaluation of Educational Quality
